# Etymologia: Dermatophyte

**DOI:** 10.3201/eid2609.ET2609

**Published:** 2020-09

**Authors:** Aline E. Santana, Fábio P. Sellera

**Affiliations:** Universidade de São Paulo, São Paulo, Brazil

**Keywords:** dermatophyte, dermatophytosis, fungi, pathogenic fungus, Raymond Jacques Adrien Sabouraud, zoonoses

## Dermatophyte [dur′mə-to-fit′′]

From the Greek *derma* (skin) + *phyton* (plant), dermatophytes are a group of 3 genera of filamentous fungi (*Microsporum*, *Epidermophyton*, and *Trichophyton*) that have the ability to invade keratinized tissues and cause superficial infections in humans and animals (Figure). Dermatophytes were improperly assigned to the Plantae kingdom until 1969, when they were then classified into the Fungi kingdom.

**Figure:**
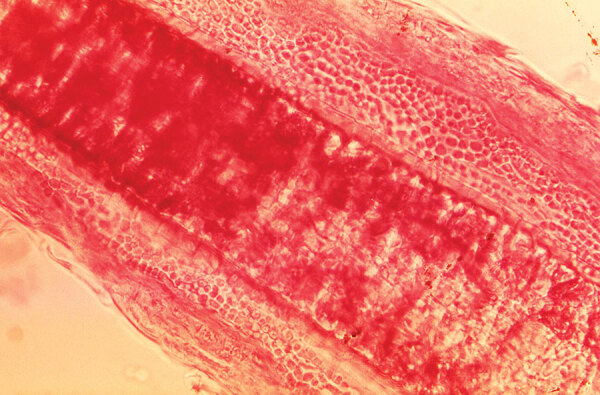
This photomicrograph of a guinea pig hair shaft specimen revealed ultrastructural features exhibited at the site of a ringworm infection by the dermatophyte, *Trichophyton mentagrophytes*. Note that the sporangia were confined to the outer region of the hair shaft, known as an exothrix infection. CDC/Dr. Lucille K. Georg, 1968. Original magnification 430×,

Dermatophytosis is also referred to as ringworm or tinea (Latin for “worm”) because it can cause ring-shaped patches that are usually red, itchy, and have worm-like borders. In 1910, Raymond Jacques Adrien Sabouraud, a French dermatologist, was the first to report the morphologic characteristics of dermatophytes. During the decades that followed, taxonomy of dermatophytes has gone through revolutionary changes, mostly due to the advent of molecular diagnosis. Although studies performed in the 21st century have resulted in further classification changes and consolidation of new species, debates regarding the taxonomy of dermatophyte agents persist.
